# Supplementary material for: Comparison of Early Contrast Enhancement Models in Ultrafast Dynamic Contrast-Enhanced Magnetic Resonance Imaging of Prostate Cancer
Source: Diagnostics (Basel). 2024 Apr 23;14(9):870. doi: 10.3390/diagnostics14090870 (PMC11083228; doi:10.3390/diagnostics14090870)
Supplement: Supplementary file 1 [file diagnostics-14-00870-s001.zip › diagnostics-2892195-supplementary.pdf]

Table S1. Post-hoc analysis of differences within PI_RADS and prostate zone showing differences in ANOVA

Parameter (Model)	Factors	Difference	lower 95%CI	upper 95% CI	p-value
F_p (2CU)	P3-Normal	0.3677	-1.2193	1.9548	0.9282
	P4-Normal	1.2499	0.4199	2.0799	0.0010
	P5-Normal	1.8516	0.7734	2.9299	0.0002
	P4-P3	0.8822	-0.7582	2.5226	0.4929
	P5-P3	1.4839	-0.2950	3.2629	0.1342
	P5-P4	0.6017	-0.5536	1.7570	0.5204
	TZ-PZ	0.7450	0.0227	1.4673	0.0434
MTT_p (2CU)	P3-Normal	-0.4264	-1.0500	0.1971	0.2811
	P4-Normal	-0.4775	-0.8036	-0.1514	0.0015
	P5-Normal	-0.6180	-1.0416	-0.1945	0.0015
	P4-P3	-0.0510	-0.6955	0.5935	0.9967
	P5-P3	-0.1916	-0.8905	0.5073	0.8876
	P5-P4	-0.1406	-0.5945	0.3133	0.8464
	TZ-PZ	-0.2030	-0.4868	0.0808	0.1579
T_c (2CU)	P3-Normal	-0.4542	-1.1186	0.2101	0.2813
	P4-Normal	-0.4985	-0.8459	-0.1510	0.0019
	P5-Normal	-0.6670	-1.1183	-0.2157	0.0013
	P4-P3	-0.0442	-0.7309	0.6424	0.9982
	P5-P3	-0.2128	-0.9574	0.5319	0.8749
	P5-P4	-0.1685	-0.6522	0.3151	0.7948
	TZ-PZ	-0.2360	-0.5384	0.0663	0.1238
α (Exponential EMM)	P3-Normal	0.0157	-0.0499	0.0812	0.9218
	P4-Normal	0.0675	0.0332	0.1017	1.3×10^{-5}
	P5-Normal	0.0899	0.0454	0.1344	8.0×10^{-6}
	P4-P3	0.0518	-0.0160	0.1195	0.1929
	P5-P3	0.0742	0.0008	0.1477	0.0468
	P5-P4	0.0224	-0.0253	0.0701	0.6041
	TZ-PZ	0.0464	0.0166	0.0762	0.0028
A_1-T_0 (Sigmoidal EMM)	P3-Normal	-6.3268	-13.9347	1.2811	0.1361
	P4-Normal	-7.8307	-11.8094	-3.8519	1.32×10^{-5}
	P5-Normal	-7.6588	-12.8274	-2.4903	1.26×10^{-3}
	P4-P3	-1.5039	-9.3676	6.3598	0.9578
	P5-P3	-1.3321	-9.8597	7.1956	0.9762
	P5-P4	0.1718	-5.3663	5.7100	0.9998
	TZ-PZ	-3.8757	-7.3381	0.4133	0.02883
A_2 (Sigmoidal EMM)	P3-Normal	-6.0319	-13.3057	1.2420	0.1378
	P4-Normal	-7.6446	-11.4486	-3.8405	8.8×10^{-6}
	P5-Normal	-6.7672	-11.7088	-1.8256	0.0033
	P4-P3	-1.6127	-9.1311	5.9057	0.9419
	P5-P3	-0.7354	-8.8886	7.4179	0.9952
	P5-P4	0.8773	-4.4177	6.1724	0.9719
	TZ-PZ	-3.5725	-6.8828	-0.2621	0.0349
TTP	P3-Normal	-1.7669	-30.3493	26.8156	0.9984
	P4-Normal	-29.0135	-43.9614	-14.0655	1.73×10^{-5}
	P5-Normal	-36.7717	-56.1896	-17.3537	2.76×10^{-5}
	P4-P3	-27.2466	-56.7901	2.2969	0.0811
	P5-P3	-35.0048	-67.0428	-2.9668	0.0269
	P5-P4	-7.7582	-28.5649	13.0485	0.7595
	TZ-PZ	-3.3312	-16.3393	9.6769	0.6108

Table S2. Post-hoc analysis of differences within Grade Group following one-way ANOVA

Parameter (Model)	Factors	Difference	lower 95%CI	upper 95% CI	p-value
F_P	GG 1 vs -ve or Normal-appearing	1.096	-0.352	2.545	0.22006
(2CU)	GG 2 vs -ve or Normal-appearing	1.329	0.144	2.515	0.02064
	GG 3 vs -ve or Normal-appearing	1.932	0.331	3.532	0.01056
	GG 4 vs -ve or Normal-appearing	2.017	0.417	3.617	0.00683
	GG 2 vs GG 1	0.233	-1.500	1.966	0.99544
	GG 3 vs GG 1	0.835	-1.204	2.875	0.77563
	GG 4 vs GG 1	0.921	-1.119	2.960	0.70761
	GG 3 vs GG 2	0.602	-1.260	2.464	0.89059
	GG 4 vs GG 2	0.687	-1.174	2.549	0.83434
	GG 4 vs GG 3	0.085	-2.064	2.235	0.99996
MTT_P	GG 1 vs -ve or Normal-appearing	-0.600	-1.208	0.007	0.05411
(2CU)	GG 2 vs -ve or Normal-appearing	-0.428	-0.925	0.069	0.12214
	GG 3 vs -ve or Normal-appearing	-0.617	-1.288	0.054	0.08542
	GG 4 vs -ve or Normal-appearing	-0.582	-1.253	0.088	0.11772
	GG 2 vs GG 1	0.172	-0.555	0.899	0.96233
	GG 3 vs GG 1	-0.017	-0.872	0.838	1.00000
	GG 4 vs GG 1	0.018	-0.837	0.873	1.00000
	GG 3 vs GG 2	-0.189	-0.969	0.592	0.95945
	GG 4 vs GG 2	-0.154	-0.935	0.627	0.98057
	GG 4 vs GG 3	0.035	-0.867	0.936	0.99997
T_c	GG 1 vs -ve or Normal-appearing	-0.631	-1.277	0.015	0.05851
(2CU)	GG 2 vs -ve or Normal-appearing	-0.467	-0.996	0.061	0.10651
	GG 3 vs -ve or Normal-appearing	-0.672	-1.385	0.041	0.07397
	GG 4 vs -ve or Normal-appearing	-0.627	-1.340	0.087	0.11049
	GG 2 vs GG 1	0.164	-0.609	0.936	0.97484
	GG 3 vs GG 1	-0.041	-0.950	0.868	0.99994
	GG 4 vs GG 1	0.004	-0.905	0.913	1.00000
	GG 3 vs GG 2	-0.204	-1.034	0.626	0.95656
	GG 1 vs -ve or Normal-appearing	-0.159	-0.989	0.671	0.98246
	GG 2 vs -ve or Normal-appearing	0.045	-0.913	1.004	0.99993
E	GG 1 vs -ve or Normal-appearing	-0.00681	-0.04878	0.03515	0.99064
(2CU)	GG 2 vs -ve or Normal-appearing	-0.02546	-0.05980	0.00888	0.23804
	GG 3 vs -ve or Normal-appearing	-0.03603	-0.08239	0.01033	0.19764
	GG 4 vs -ve or Normal-appearing	-0.03551	-0.08187	0.01085	0.20964
	GG 2 vs GG 1	-0.01865	-0.06886	0.03157	0.83143
	GG 3 vs GG 1	-0.02921	-0.08830	0.02987	0.63273
	GG 4 vs GG 1	-0.02870	-0.08778	0.03039	0.64818
	GG 3 vs GG 2	-0.01057	-0.06450	0.04337	0.98107
	GG 1 vs -ve or Normal-appearing	-0.01005	-0.06399	0.04389	0.98428
	GG 2 vs -ve or Normal-appearing	0.00052	-0.06176	0.06280	0.99999

Table S2 - continued. Post-hoc analysis of differences within Grade Group following one-way ANOVA

	Factors	Difference	lower 95%CI	upper 95% CI	p-value
α (exponential EMM)	GG 1 vs -ve or Normal-appearing	0.0728	0.0137	0.1320	0.00866
	GG 2 vs -ve or Normal-appearing	0.0580	0.0096	0.1063	0.01141
	GG 3 vs -ve or Normal-appearing	0.0689	0.0035	0.1342	0.03413
	GG 4 vs -ve or Normal-appearing	0.0769	0.0116	0.1422	0.01340
	GG 2 vs GG 1	-0.0149	-0.0857	0.0559	0.97540
	GG 3 vs GG 1	-0.0040	-0.0873	0.0793	0.99992
	GG 4 vs GG 1	0.0041	-0.0792	0.0873	0.99992
	GG 3 vs GG 2	0.0109	-0.0651	0.0869	0.99416
	GG 4 vs GG 2	0.0190	-0.0571	0.0950	0.95462
	GG 4 vs GG 3	0.0081	-0.0797	0.0958	0.99898
$A_1 - T_0$ (sigmoidal EMM)	GG 1 vs -ve or Normal-appearing	-3.973	-12.262	4.316	0.65939
	GG 2 vs -ve or Normal-appearing	-5.809	-12.592	0.974	0.12632
	GG 3 vs -ve or Normal-appearing	-8.390	-17.546	0.766	0.08728
	GG 4-0 -ve or Normal-appearing	-7.765	-16.922	1.391	0.13266
	GG 2 vs GG 1	-1.835	-11.753	8.082	0.98468
	GG 3 vs GG 1	-4.417	-16.087	7.253	0.82158
	GG 4 vs GG 1	-3.792	-15.462	7.878	0.88886
	GG 3 vs GG 2	-2.581	-13.234	8.072	0.95903
	GG 4 vs GG 2	-1.957	-12.610	8.696	0.98511
	GG 4 vs GG 3	0.625	-11.677	12.926	0.99990
A2 (sigmoidal EMM)	GG 1 vs -ve or Normal-appearing	-2.827	-10.635	4.981	0.84386
	GG 2 vs -ve or Normal-appearing	-5.742	-12.132	0.647	0.09753
	GG 3 vs -ve or Normal-appearing	-7.853	-16.478	0.772	0.09057
	GG 4 vs -ve or Normal-appearing	-7.755	-16.380	0.870	0.09730
	GG 2 vs GG 1	-2.915	-12.257	6.427	0.90259
	GG 3 vs GG 1	-5.026	-16.019	5.967	0.69780
	GG 4 vs GG 1	-4.928	-15.921	6.065	0.71294
	GG 3 vs GG 2	-2.111	-12.146	7.925	0.97542
	GG 4 vs GG 2	-2.013	-12.048	8.023	0.97936
	GG 4 vs GG 3	0.098	-11.490	11.686	0.99999
TTP	GG 1 vs -ve or Normal-appearing	-33.491	-58.461	-8.522	0.00344
	GG 2 vs -ve or Normal-appearing	-21.986	-42.419	-1.553	0.02903
	GG 3 vs -ve or Normal-appearing	-36.059	-63.641	-8.476	0.00460
	GG 4-0 -ve or Normal-appearing	-39.026	-66.608	-11.443	0.00181
	GG 2 vs GG 1	11.505	-18.370	41.381	0.81215
	GG 3 vs GG 1	-2.567	-37.721	32.587	0.99958
	GG 4 vs GG 1	-5.534	-40.688	29.620	0.99168
	GG 3 vs GG 2	-14.072	-46.164	18.019	0.72920
	GG 4 vs GG 2	-17.039	-49.131	15.052	0.56744
	GG 4 vs GG 3	-2.967	-40.023	34.089	0.99940
